# Young MSM changed temporal HIV-1 epidemic pattern in Heilongjiang Province, China

**DOI:** 10.3389/fmicb.2022.1028383

**Published:** 2022-11-25

**Authors:** Qing-Hai Li, Jia-Ye Wang, Si-Yu Liu, Yun-Qi Zhang, En-Long Li, Yi-Ru Wang, Shu-Lei Zhang, Wen-Bo Zhao, Shu-Lin Liu, Xiao-Hong Chen, Fu-Xiang Wang

**Affiliations:** ^1^Genomics Research Center, College of Pharmacy, Harbin Medical University, Harbin, China; ^2^Department of Microbiology, Harbin Medical University, Harbin, China; ^3^Department of Infectious Diseases, The Fourth Affiliated Hospital of Harbin Medical University, Harbin, China; ^4^Department of Infectious Diseases, The Second Affiliated Hospital of Southern University of Science and Technology, Shenzhen, China; ^5^Department of Infectious Diseases, The Third People’s Hospital of Shenzhen, Shenzhen, China

**Keywords:** HIV-1 epidemic, young men who have sex with men, CRF01_AE, immune status, recent infection

## Abstract

**Background:**

Human immunodeficiency virus type 1 (HIV-1) epidemic in China is featured by geographical diversity of epidemic patterns. Understanding the characteristics of regional HIV-1 epidemic allows carrying out targeted prevention and controlling measures. This seven-year cross-sectional study was conducted in Heilongjiang, one province of Northeast China, where newly diagnosed infection is fast increasing yearly, but temporal HIV-1 epidemic trend is largely unknown.

**Methods:**

Information of 1,006 newly diagnosed HIV-1-infected participants were collected before antiretroviral therapy during 2010–2016 in Heilongjiang province. HIV-1 genotype was identified based on the viral *gag* and *env* gene sequences. Recent infection was determined by Limiting-Antigen Avidity assays. Comparison analyses on the median ages, CD4 counts, proportions of stratified age groups and CD4 count groups, and rates of recent HIV-1 infection among different population and sampling times were performed to understand temporal HIV-1 epidemic features.

**Results:**

Homosexual contact among men who have sex with men (MSM) was the main transmission route and CRF01_AE was the most dominant HIV-1 genotype. During 2010–2016, the HIV-1 epidemic showed three new changes: the median age continued to decline, the cases with a CD4 count more than 500 cells/μl (CD4hi cases) disproportionally expanded, and the recent HIV-1 infection rate steadily increased. MSM cases determined the temporal trend of HIV-1 epidemic here. Increase of young MSM cases (aged <30 years) made the main contribution to the younger age trend of MSM cases. These young MSM exhibited a higher median CD4 count, a higher proportion of CD4hi cases, and a higher rate of recent HIV-1 infection than cases aged 30 years and more. MSM infected by CRF01_AE virus mostly affected HIV-1 epidemic patterns among MSM population.

**Conclusion:**

Young MSM have become a new hotspot and vulnerable group for HIV-1 transmission in Heilongjiang Province, Northeast China. The rapid increase in the number of young MSM cases, mainly those with CRF01_AE infection, changed temporal HIV-1 epidemic pattern here. Measures for prevention and control of HIV-1 infection among this population are urgently needed in the future.

## Introduction

Human immunodeficiency virus/acquired immunodeficiency syndrome (HIV/AIDS) epidemic in China becomes increasingly severe and complicated. By the end of 2018, the reported absolute number of Chinese people living with HIV-1 was approximately 0.89 million, but the real number was estimated at more than 1.25 million (Wu et al., 2021). One big challenge for HIV/AIDS control is the geographical and temporal diversity of epidemic patterns throughout the whole country. In the past 20 years, HIV/AIDS epidemic pattern has obviously changed, including regional distribution, transmission route, age composition, and dominant HIV-1 genotypes (Liu et al., 2018; Ding et al., 2019; Wu et al., 2019). For the regions with high HIV-1 incidence such as southwest provinces Yunnan and Guangxi, the features of HIV/AIDS epidemic have been described extensively (Wu et al., 2019; Chen et al., 2019a, 2019b). However, for the regions where HIV-1 incidence is relatively low but annually reported case number rises rapidly, such as Heilongjiang Province, the data about HIV/AIDS epidemic are limited and sporadic.

Heilongjiang, one province in Northeast China, lies on the border between China and Russia. Since first HIV-1 infection was identified in 1993, annually reported HIV-1 diagnoses in Heilongjiang continued to increase slowly until the year 2008 when an expansion of HIV-1 infection among men who have sex with men (MSM) occurred (Shao et al., 2014). Since then, newly diagnosed HIV/AIDS cases rapidly increased yearly, and homosexual contact among MSM became dominant route of HIV-1 transmission in this province. By the end of 2017, there were 9,495 people living with HIV-1 in Heilongjiang (Ma et al., 2018). MSM accounted for 64.4% of newly reported cases in 2010–2011 and 72.0% in 2012–2017 (Shao et al., 2014; Ma et al., 2018). The predominant HIV-1 genotype circulating in Heilongjiang has also changed, from subtype B to circulating recombinant form 01_AE (CRF01_AE) during 2009–2012 (Wang et al., 2007; Liu et al., 2009; Shao et al., 2013, 2016; Li et al., 2016a). Since being introduced into China in the 1990s, CRF01_AE virus has gradually evolved into several specialized clusters among specialized population in specialized geographical regions. Heilongjiang is one of the hotspots where CRF01_AE infection concentrates on MSM population (Feng et al., 2013; Li et al., 2017). However, the current epidemic trend of HIV-1 infections in this region, especially among MSM or CRF01_AE-infected MSM, is largely unknown.

In this study, we collected clinical information and samples from more than 1,000 new HIV-1 diagnoses in Heilongjiang Province and made a 7-year cross-sectional study. This paper reported that MSM was driving HIV-1 epidemic trend in Heilongjiang Province and highlighted the critical role of young MSM, especially those infected by CRF01_AE virus, on the change of HIV-1 epidemic pattern here.

## Materials and methods

### The study participants and information collection

All the participants were recruited in the Fourth Affiliated Hospital of Harbin Medical University from January 2010 to December 2016. Peripheral blood samples were collected immediately after diagnosis for HIV-1 genotyping. The information including sex, age, the way to acquire HIV-1, and peripheral blood CD4 positive T cell count (hereinafter referred to as CD4 count) at diagnosis were also collected. The inclusion criteria of the participants included: (1) being newly diagnosed as HIV-1 infection, (2) being before antiretroviral treatment, and (3) having age and CD4 count information recorded at diagnosis. Participants who lacked a peripheral blood sample or for whom HIV-1 genotype information was not available were excluded.

### HIV-1 genotyping

For each participant, 5 ml of whole peripheral blood was collected, and then, plasma sample was separated for HIV-1 genotyping. HIV-1 *gag* p17-p24 (HXB2: nt836-1,507) and *env* C2-C4 (HXB2: nt7,002-7,541) genes were amplified from the plasma samples by RT-PCR and subject to sequencing. The genotypes of *gag* and *env* genes were determined by the cluster distribution of genes in neighbor-joining trees constructed using MEGA 6.06 software as previously described (Li et al., 2019a). The *gag* and *env* genotyping information were used together to characterize the viral genotype. For samples with only one *gag* or *env* gene, the viral genotype was determined by the available single-gene genotyping. Considering that the *gag* and *env* regions of CRF07_BC, CRF08_BC, or subtype C virus analyzed in this study were all subtype C, for the sample with *gag* or *env* gene from CRF07_BC, CRF08_BC, or subtype C, the genotype of the single gene was named as 07&08&C. For the sample with discordant genotypes of *env* and *gag* genes or with an inter-subtype recombinant *gag* gene, the genotype of HIV-1 virus was identified as unique recombinant form (URF).

### HIV-1 infection status evaluation

The recent or long-term infection of each participant was determined by the Limiting-Antigen Avidity assays using HIV-1 LAg-Avidity EIA kit (Beijing Kinghawk Pharmaceutical Co., Ltd., Beijing, China) as previously described (Duong et al., 2012; Li et al., 2019a). A normalized optical density (ODn) value of 1.5 was used as the cutoff to determine HIV-1 infection status. Samples with ODn values ranging from 0.4 to 1.5 (including 1.5) were classified as recent infections, and those with values above 1.5 were classified as long-term infections. The recent infection indicated that the individual acquired HIV-1 within a mean time interval of 130 days.

### Genbank accession numbers of nucleotide sequences

The nucleotide sequences identified in this study were submitted to GenBank with accession numbers of MK702086-MK702931 for *gag* p17-p24 genes and MK702932-MK703795 for *env* C2-C4 genes.

### Statistical analyses

Sampling years were divided into three time phases: 2010–2011, 2012–2014, and 2015–2016. Mann–Whitney test and Kruskal–Wallis test were used for comparison of the quantitative variables across two groups and multiple groups, respectively. Chi-square test was used for comparison of qualitative variables across groups. Nonparametric Spearman correlation test was used to analyze the relationship between variables. Data analyses were performed by GraphPad Prism version 8.3.0 (GraphPad Software, Inc., La Jolla, CA). The *p*-value less than 0.05 was considered as significant unless otherwise specified.

## Results

### Basic information

From 2010 to 2016, 1,183 newly diagnosed HIV-1 cases were collected. Of them, 177 participants were excluded because of lack of blood samples or unavailability of HIV-1 genotype information. Finally, 1,006 cases were included in our subsequent analyses. In order to ensure the exclusion of participants did not introduce a bias in data analysis, the basic information between the included cases and the excluded cases were compared. There was no significant difference in the median age, CD4 cell count, gender composition, and risk group composition between the two groups (Supplementary Table S1).

Of the 1,006 participants in this study, the majority were male, accounting for 93.7%. Median age was 35 years (interquartile range [IQR] 29–45), and median CD4 count was 384 cells/μl (IQR 214–490). The most important mode of HIV-1 transmission was male-to-male sexual contact among MSM, followed by heterosexual contact. These two modes of sexual contacts together accounted for 85.7% (862/1006) of all cases and 98.6% (862/874) of cases with known transmission routes.

From 1,006 plasma samples, 864 *env* genes (including 543 CRF01_AE, 172 subtype B, 144 07&08&C and 5 subtype A) and 846 *gag* genes (including 567 CRF01_AE, 134 subtype B, 123 07&08&C, 6 subtype A and 16 inter-subtype recombinants) were obtained. That was, 704 samples had both *env* and *gag* genes, 160 samples had *env* gene alone and 142 samples had *gag* gene alone (Supplementary Figure S1). The most prevalent genotype of HIV-1 viruses was CRF01_AE (accounting for 62.5% of all cases), followed by subtype B (16.5%) and 07&08&C (13.0%). In addition, 73 URFs were also identified, including 57 viruses having discordant genotypes of *env* and *gag* genes and 16 viruses having an inter-subtype recombinant *gag* gene (colored in green in Supplementary Figure S1B). Seven subtype A viruses were also found (Table 1).

**Table 1:** Basic information of the participants in this study.

	Total (*n* = 1,006)	Sampling year			*χ* ^2^	*p*-value
		2010–2011 (*n* = 140)	2012–2014 (*n* = 299)	2015–2016 (*n* = 567)		
Sex					1.09	0.5794
Male	943 (93.7)	134 (95.7)	279 (93.3)	530 (93.5)	1.09	0.5794
Female	63 (6.3)	6 (4.3)	20 (6.7)	37 (6.5)	1.09	0.5794
Age (years old)					18.04	0.0061
<30	275 (27.3)	23 (16.4)	86 (28.8)	166 (29.3)	9.77	0.0076
30–39	349 (34.7)	52 (37.1)	94 (31.4)	203 (35.8)	2.08	0.3540
40–49	215 (21.4)	37 (26.4)	57 (19.1)	121 (21.3)	3.08	0.2145
>49	167 (16.6)	28 (20.0)	62 (20.7)	77 (13.6)	8.60	0.0136
CD4 count (cells/μl)					12.95	0.0438
<200	227 (22.5)	29 (20.7)	69 (23.1)	129 (22.8)	0.33	0.8477
200–350	194 (19.3)	35 (25.0)	63 (21.1)	96 (16.9)	5.57	0.0618
351–500	345 (34.3)	53 (37.9)	105 (35.1)	187 (33.0)	1.31	0.5187
>500	240 (23.9)	23 (16.4)	62 (20.7)	155 (27.3)	9.64	0.0081
Risk group					39.87	< 0.0001
MSM	667 (66.3)	82 (58.6)	199 (66.6)	386 (68.1)	4.55	0.1026
Heterosexual	195 (19.4)	37 (26.4)	64 (21.4)	94 (16.6)	8.08	0.0176
Other groups	12 (1.2)	8 (5.7)	2 (0.7)	2 (0.4)	28.38	< 0.0001
Unknown	132 (13.1)	13 (9.3)	34 (11.4)	85 (15.0)	4.35	0.1137
HIV-1 genotype (*gag*/*env*)					31.19	0.0001
CRF01_AE	629 (62.5)	75 (53.6)	198 (66.2)	356 (62.8)	6.55	0.0378
Subtype B	166 (16.5)	42 (30.0)	48 (16.1)	76 (13.4)	22.51	< 0.0001
07&08&C	131 (13.0)	18 (12.9)	36 (12.0)	77 (13.6)	0.41	0.8131
Subtype A	7 (0.7)	0 (0.0)	1 (0.3)	6 (1.1)	NA	NA
URF	73 (7.3)	5 (3.6)	16 (5.4)	52 (9.2)	7.53	0.0232
HIV-1 infection status					12.19	0.0023
Recent infection	367 (36.5)	38 (27.1)	97 (32.4)	232 (40.9)	12.19	0.0023
Long-term infection	639 (63.5)	102 (72.9)	202 (67.6)	335 (59.1)	12.19	0.0023

07&08&C, HIV-1 virus that had a genotype of CRF07_BC, CRF08_BC or subtype C; MSM, men who have sex with men; NA: not applicable; other groups, risk groups including former plasma donors (8 cases), injection drug users (3 cases) and mother-to-child infection (1 case); URF, unique recombinant form. Both *gag* and *env* genes were used together to determine HIV-1 genotype of one sample. For the sample with only one gene available, HIV-1 genotype was determined by the known gene. Data were shown as number (%). The comparisons of the proportions among three sampling times were done by Chi-square test with *p* < 0.05 as significant. The Post hoc multiple comparisons were done with *p* < 0.0125 as significant for age groups, CD4 count groups, risk groups, and HIV-1 genotype groups.

### MSM cases determined the temporal trend of HIV-1 epidemic

**Figure 1:**
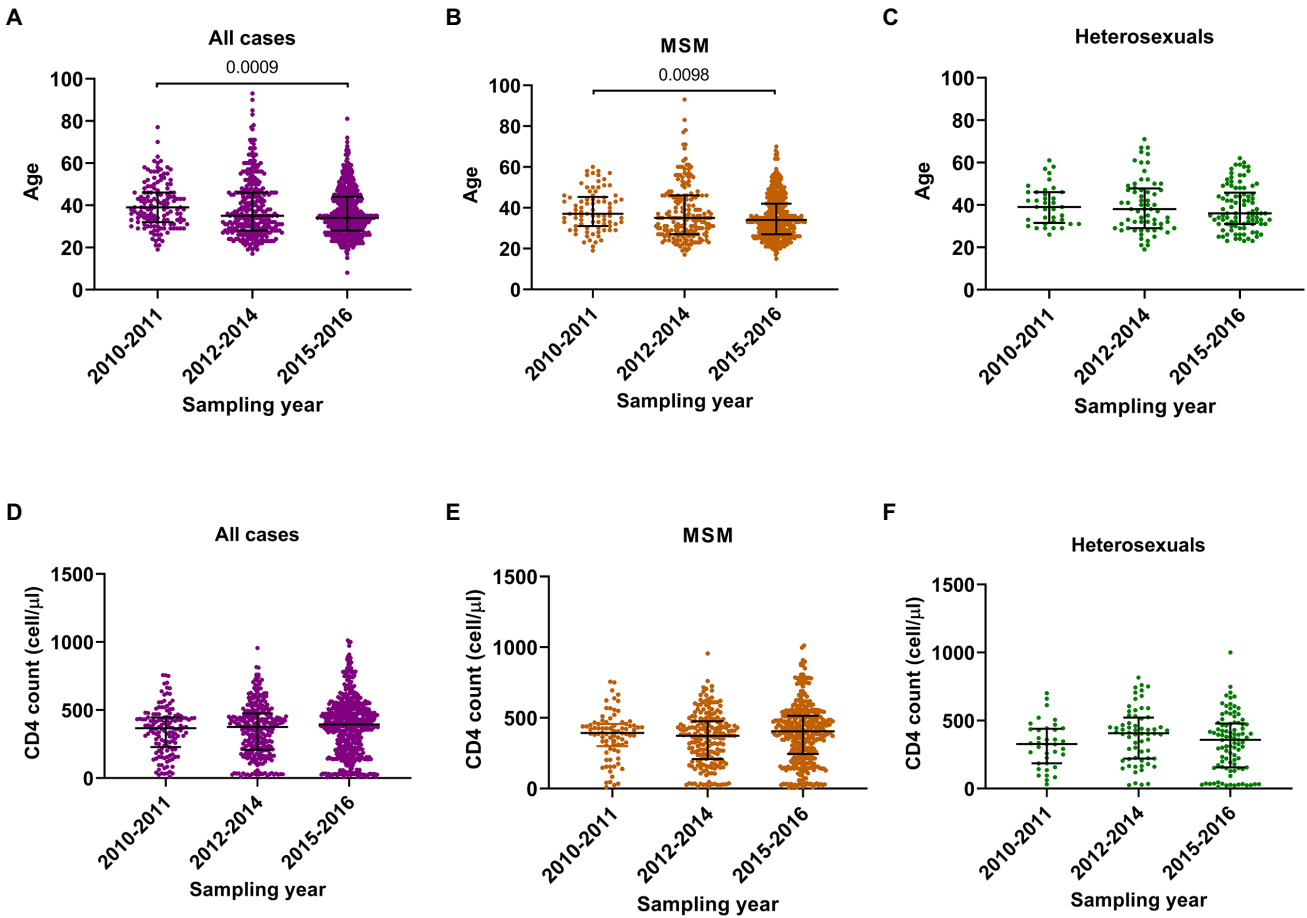
Comparison analyses on ages and CD4 cell counts in three sampling times. **(A–C)** The age in all participants, men who have sex with men (MSM) and heterosexuals. **(D–F)** The CD4 cell count in all participants, MSM and heterosexuals. Data were shown as median with interquartile range. Comparison across groups was done by Kruskal–Wallis test.

During the three sampling times, three main changes in HIV-1 epidemic were found. Firstly, the median age declined from 39 years (IQR 32–46) to 34 years (IQR 28–44) (Figure 1A), suggesting a younger age trend of these cases. Secondly, although the median CD4 counts did not change significantly (Figure 1D), the proportion of cases with CD4 counts more than 500 cells/μl (hereinafter referred to as CD4hi cases) arose gradually from 16.4 to 27.3% (Table 1), indicating the expansion of cases with good immune status. Thirdly, the rate of recent HIV-1 infection gradually increased from 27.1% to 40.9% (Table 1), suggesting the increase of cases who were diagnosed at the early stage of HIV-1 infection.

The changes observed for the entire group were similar among MSM cases: median age decreased from 37 years (IQR 31–45) to 34 years (IQR 27–42) (Figure 1B), the proportion of CD4hi cases increased from 15.9% to 28.8%, and the recent infection rate increased from 32.9% to 43.5% (Table 2; Figure 1E). But such changes were not seen in heterosexual cases (Figures 1C,F; Supplementary Table S2). These data indicated that MSM cases dominated the trend of HIV-1 epidemic among the newly diagnosed infections, and therefore, understanding the contributors to changes in HIV-1 epidemic patterns among MSM cases would be helpful to find out key factors for monitoring and controlling HIV-1 infection in this region.

**Table 2:** Basic information of the HIV-1 infected MSM cases.

	Total (*n* = 667)	Sampling year			*χ* ^2^	*p*-value
		2010–2011 (*n* = 82)	2012–2014 (*n* = 199)	2015–2016 (*n* = 386)		
Age (years old)					10.89	0.0919
<30	204 (30.6)	17 (20.7)	63 (31.7)	124 (32.1)		
30–39	244 (36.5)	29 (35.4)	68 (34.2)	147 (38.1)		
40–49	125 (18.7)	21 (25.6)	33 (16.6)	71 (18.4)		
>49	94 (14.1)	15 (18.3)	35 (17.6)	44 (11.4)		
CD4 count (cells/μl)					13.70	0.0332
<200	135 (20.2)	13 (15.9)	45 (22.6)	77 (19.9)	1.69	0.4292
200–350	125 (18.7)	16 (19.5)	43 (21.6)	66 (17.1)	1.79	0.4080
351–500	243 (36.4)	40 (48.8)	71 (35.7)	132 (34.2)	6.28	0.0433
>500	164 (24.6)	13 (15.9)	40 (20.1)	111 (28.8)	9.15	0.0103
HIV-1 genotype (*gag*/*env*)					15.06	0.0198
CRF01_AE	429 (64.3)	44 (53.7)	135 (67.8)	250 (64.8)	5.17	0.0754
Subtype B	98 (14.7)	22 (26.8)	30 (15.1)	46 (11.9)	12.03	0.0024
07&08&C	86 (12.9)	11 (13.4)	21 (10.6)	54 (14.0)	1.40	0.4957
Subtype A & URF	54 (8.1)	5 (6.1)	13 (6.5)	36 (9.3)	1.88	0.3908
HIV-1 infection status					8.51	0.0142
Recent infection	259 (38.8)	27 (32.9)	64 (32.2)	168 (43.5)	8.51	0.0142
Long-term infection	408 (61.2)	55 (67.1)	135 (67.8)	218 (56.5)	8.51	0.0142

07&08&C, HIV-1 virus that had a genotype of CRF07_BC, CRF08_BC or subtype C; MSM, men who have sex with men; URF, unique recombinant form. Both *gag* and *env* genes were used together to determine HIV-1 genotype of one sample. For the sample with only one gene available, HIV-1 genotype was determined by the known gene. Data were shown as number (%). The comparisons of the proportions among three sampling times were done by Chi-square test with P < 0.05 as significant. The Post hoc multiple comparisons were done with *p* < 0.0125 as significant for CD4 count groups and HIV-1 genotype groups.

### Increase of young MSM cases made the main contribution to the decline in median age of HIV-1-infected MSM

Median age of all MSM cases was 34 years (IQR 28–44). Cases aged <30 years (hereinafter referred to as young cases), 30–39 years, 40–49 years, and above 49 years accounted for 30.6%, 36.5%, 18.7%, and 14.1% of MSM cases, respectively. From 2010–2011 to 2015–2016, the proportion of young cases increased from 20.7% (17/82) to 32.1% (124/386, *p* = 0.0411), while the proportions of other age groups did not significantly change (Table 2), implying that the increase of young MSM cases was the main cause for the younger age trend of HIV-1-infected MSM.

### Young MSM cases exhibited better immune state and a higher rate of recent HIV-1 infection

**Figure 2:**
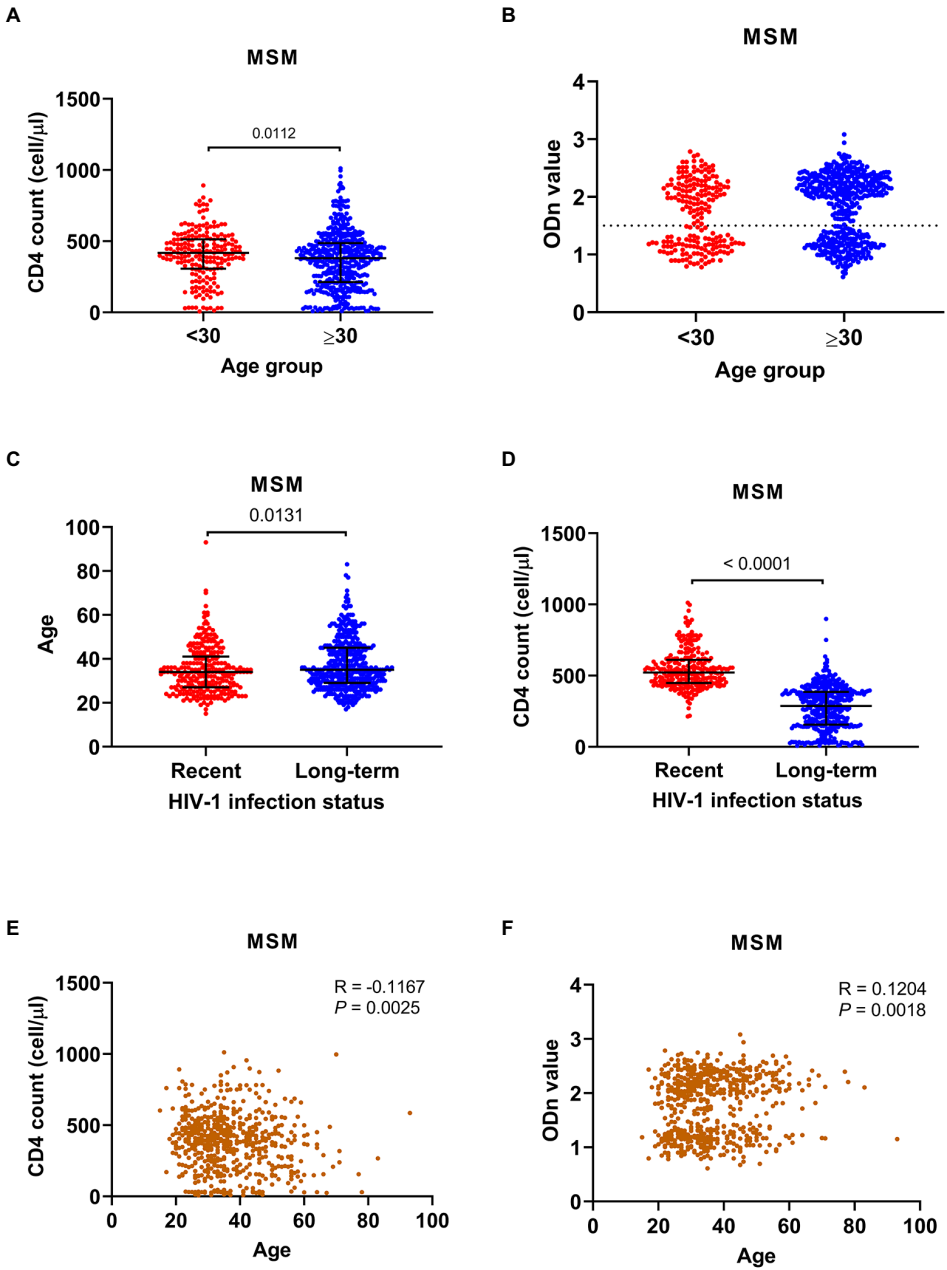
Comparison analyses between age groups and HIV-1 infection status groups of men who have sex with men (MSM). **(A)** The CD4 cell count in cases aged **<**30 years and those aged ≥30 years. **(B)** The normalized optical density (ODn) value of plasma samples from cases aged **<**30 years and cases aged ≥30 years in Limiting-Antigen Avidity assays. **(C)** The ages of MSM with recent and long-term HIV-1 infections. **(D)** The CD4 cell count of MSM with recent and long-term HIV-1 infections. **(E,F)** Correlation analyses between age and CD4 cell count and ODn value. Data in **(A–D)** were shown as median with interquartile range, and the comparison across groups was done by Kruskal–Wallis test. The correlation analyses in **(E,F)** were done by nonparametric Spearman correlation test.

Compared to MSM cases aged ≥30 years (hereinafter referred to as old cases), young MSM cases had a higher median CD4 count (418 [IQR 307-514] versus 381 [IQR 213–487]) (Figure 2A). Additionally, 28.9% (59/204) of young MSM were CD4hi cases, slightly higher than that of old cases (22.7%, 105/463, *p* = 0.0845), and 15.2% (31/204) of young MSM had a CD4 count less than 200 cells/μl, lower than that of old cases (22.5%, 104/463, *p* = 0.0314).

The recent infection rate of all MSM cases was 38.8% (Table 2). Totally, 44.6% (91/204) of young MSM were identified as recent infections, higher than that of old MSM (36.3%, 168/463, *p* = 0.0421) (Figure 2B). On the other hand, median age of recently infected MSM was lower than that of long-term infected MSM (34 [IQR 27–41] versus 35 [IQR 29–45]), and the proportion of young MSM in recent infections was higher than that in long-term infections (91/259 versus 113/408, *p* = 0.0421) (Figure 2C). As expected, the median CD4 count of recently infected MSM was much higher than that of long-term infected MSM (522 [IQR 448–610] versus 286.5 [IQR 155–386]) (Figure 2D). Of recently infected MSM, 57.5% (149/259) were CD4hi cases, much higher than that of long-term infected MSM (3.7%, 15/408, *p* < 0.0001). Of note, among all CD4hi MSM cases, 90.9% (149/164) were identified as recent infections. Correlation analysis on MSM population showed a negatively correlation between age and CD4 count and a positive correlation between age and the ODn value (Figures 2E,F).

The data above suggested that young MSM seemed to be in better immune state and have a higher recent infection rate, and that the increase of young MSM cases during the study period may be result in increase of diagnoses in early stage of HIV-1 infection and the improvement in the overall immune status of MSM cases.

### MSM infected by CRF01_AE virus mostly affected HIV-1 epidemic patterns among MSM population

Next, we wanted to know whether temporal HIV-1 epidemic trend in MSM cases was associated with the infection of a particular viral genotype.

**Figure 3:**
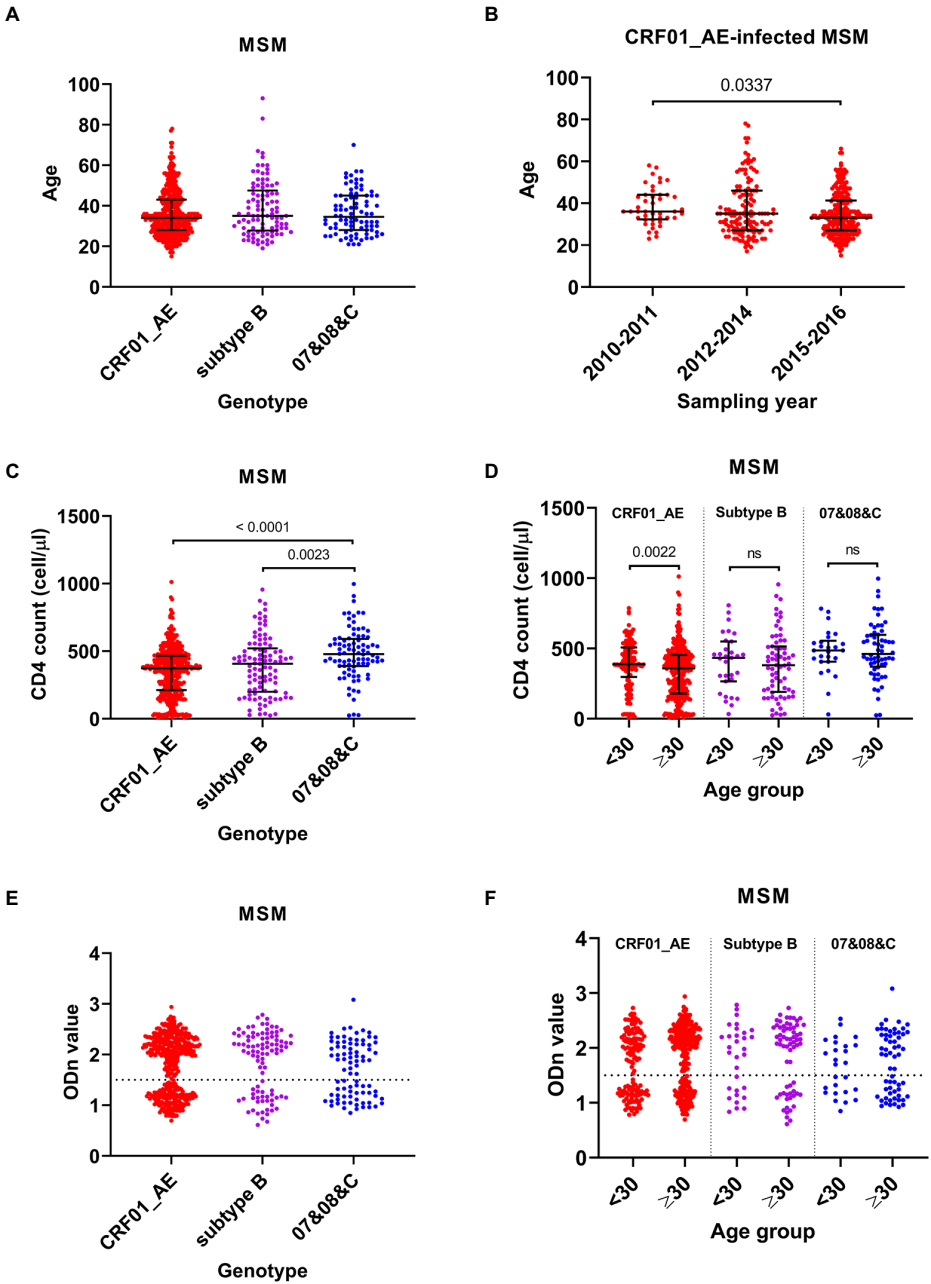
Comparison analyses among men who have sex with men (MSM) infected by different HIV-1 genotypes. **(A)** The age of MSM infected by different genotypes. **(B)** The age of CRF01_AE-infected MSM in three sampling times. **(C)** The CD4 cell count of MSM infected by CRF01_AE, subtype B, and 07&08&C viruses. **(D)** The CD4 cell count of cases aged <30 years and aged ≥30 years with different HIV-1 genotype infection. **(E)** The normalized optical density (ODn) value of plasma samples from MSM infected by different genotypes in Limiting-Antigen Avidity assays. **(F)** The ODn value of plasma samples from MSM aged <30 years and aged ≥30 years with different HIV-1 genotype infection. Data in **(A–D)** were shown as median with interquartile range, and the comparison across groups was done by Kruskal–Wallis test. 07&08&C, virus that had a genotype of CRF07_BC, CRF08_BC or subtype C.

CRF01_AE, subtype B, and 07&08&C were the main genotypes circulating among MSM cases, accounting for 64.3%, 14.7%, and 12.9% of infections, respectively (Table 2). There was no significant difference on the median ages of MSM infected by these HIV-1 genotypes (Figure 3A), but during the three sampling times, MSM cases infected by CRF01_AE virus showed similar changes with total MSM cases in general: median age decreased from 36 years (IQR 32–44) in 2010–2011 to 33 years (IQR 27–41) in 2015–2016 (Figure 3B), and the proportion of young cases increased from 13.6% (6/44) to 34.0% (85/250, *p* = 0.0071).

Median CD4 count in MSM cases infected by CRF01_AE or subtype B virus was lower than that infected by 07&08&C virus (Figure 3C). When compared with the corresponding old cases, the young MSM with CRF01_AE virus showed a higher median CD4 count (388 [IQR 297–507] versus 359 [180–453]) (Figure 3D). Furthermore, for MSM infected by CRF01_AE virus, 25.6% (34/133) of young cases were CD4hi cases, higher than that of old cases (14.9%, 44/296, *p* = 0.0079), and 15.0% (20/133) of young cases had CD4 counts less than 200 cells/μl, lower than that of old cases (27.4%, 81/296, *p* = 0.0054).

There was no significant difference on the recent infection rates of MSM cases infected by CRF01_AE (37.1%, 159/429), subtype B (33.7%, 33/98), and 07&08&C (44.2%, 38/86) (Figure 3E). But young MSM infected with CRF01_AE virus showed a higher recent infection rate than its corresponding old cases (44.4% [59/133] versus 33.8% [100/296], *p* = 0.0359). Young cases and old cases in subtype B or 07&08&C infected MSM had similar rates of recent infection (Figure 3F).

These data indicated that the increase in number and proportion of young MSM infected by CRF01_AE virus made a substantial contribution to the decline in median age of MSM cases, which may lead to the improvement in the overall immune status and the increase in recent infection rate of the MSM cases. MSM infected by CRF01_AE virus mostly affected HIV-1 epidemic patterns among MSM population.

## Discussion

This study was the first cross-sectional molecular epidemiological study based on a large sample size in Heilongjiang of Northeast China. According to our previous report and the report from the Center for Disease Control and Prevention of Heilongjiang Province, during 2010–2016, 8,026 HIV-1 infection cases were newly diagnosed in this province. The participants enrolled in this study represented 12.5% (1,006/8,026) of all newly reported cases and showed representative demographic characteristics (age composition, gender composition, and risk group composition as shown in Table 1) that were seen in the provincial level (Shao et al., 2014; Ma et al., 2018). As the data released by Health Commission of Heilongjiang Province (2017) by the end of October 2017, there were 9,276 people living with HIV (PLWH), of which 51.1% were reported in Harbin City, the capital city of Heilongjiang. Recently, Heilongjiang Provincial People’s Government (2021) reported that by the end of October 2021, the cumulative number of HIV-1 infection cases reported in this province was 16,638, including 14,373 PLWH, of which 51.7% was in Harbin City. Based on these data, the participants recruited in this study represented about 25% of newly diagnosed HIV-1 cases during 2010–2016 in Harbin City.

One obvious feature of the participants in our study was that the majority (93.7%) were male, which reflected the epidemiological data from Heilongjiang Province. In the official report from the Center for Disease Control and Prevention of Heilongjiang Province, the ratio of male to female cases during 2012–2017 was 12.3:1, that was, male cases accounted for 92.5% (12.3/13.3) of new HIV-1 diagnoses (Ma et al., 2018). This phenomenon of male case polarization also exists in other regions of China where MSM population dominate newly diagnosed HIV-1 infections, such as Beijing (92.9% in 2001–2016) (Ye et al., 2020), Tianjin (96.5% in 2013–2019) (Ge et al., 2021), Nanjing of Jiangsu Province (95.0% in 2015–2017) (Li et al., 2019b), and Anhui Province (95.6% in 2011–2013) (Wu et al., 2015).

In this study, we reported that MSM cases dominated the temporal HIV-1 epidemic trend in this region: the age of cases at diagnosis was getting younger, the proportion of cases with good immune status was increasing, and the rate of recent infection was rising. We also reported that the rapid expansion of young MSM cases, especially the young MSM infected by HIV-1 CRF01_AE virus, was the key contributor to these changes.

Unlike the situation in some European and American countries where HIV-1 epidemic initiated among MSM, few cases were reported among Chinese MSM until the year 2005. Since 2010, MSM population has become the group with the highest risk for HIV-1 transmission in China (Wu et al., 2019). One obvious feature for the HIV-1 epidemic among Chinese MSM is the uneven distribution of cases across provinces and regions, highly concentrating in municipalities, provincial capitals, or cities with large population sizes and fast economic growth, such as Beijing, Shanghai, Guangzhou (Qin et al., 2017).

As the capital of Heilongjiang Province, Harbin City is one of economic centers in Northeast China as well as one of cities that were affected by the first wave of HIV-1 expansion among MSM during 2006–2008 (Qin et al., 2017). HIV-1 prevalence among MSM population in Harbin rapidly increased from 1.0 to 9.5% between 2006 and 2011 (Wang et al., 2012; Zhang et al., 2013). Our previous studies demonstrated that MSM became the highest-risk group for HIV-1 infection in Heilongjiang Province during 2009–2012, accounting for 57.9% and 69.0% of new diagnoses in 2009–2010 and 2011–2012, respectively (Shao et al., 2014). In this study, we reported that MSM cases accounted for 66.3% of all new diagnoses during 2010–2016, consistent with our previous studies (Shao et al., 2014, 2016) and similar with the cities where HIV-1-infected Chinese MSM concentrate, such as Jilin (another northeast province), Beijing (Ye et al., 2020), and Shanghai (Li et al., 2015a). Importantly, although the annual new diagnoses continued to increase, the MSM cases still accounted for a high and stable proportion (ranging from 58.6% to 68.1%) in each sampling time, suggesting a steady increase in the number of HIV-1-infected MSM cases, which could partially explain the dominant role of MSM cases on the overall HIV-1 epidemic in this region.

Young people have been considered as a hot topic in HIV-1/AIDS epidemic in China for many years. But in the initial years (2005–2008), the clustering hotspots of Chinese young people living with HIV/AIDS (aged 15–24 years) mainly distributed within heterosexuals and intravenous drug users in southwest provinces. After 2008, new hotspots among MSM in central and northeast provinces emerged. On the national scale, new HIV-1 cases aged 15–24 years increased by an annual average of 35% during 2011–2015 (Burki, 2018). For Harbin city of Heilongjiang Province, HIV/AIDS cases aged 15–24 years increased about 5-fold between 2005 and 2012 (Zhang et al., 2017). The present study showed the number of newly diagnosed young MSM (aged <30 years) had a 7.2-fold (166/23) increase from 2010–2011 to 2015–2016 and the proportion of young MSM got a 1.5-fold (from 20.7 to 32.1%) increase, implying that young MSM have become a new vulnerable group of HIV-1 infection in Heilongjiang, China. Understanding the changes on HIV-1 epidemic features accompanied by the increasing number of this young population is important and necessary.

Indeed, two interesting changes among MSM cases were also observed: steadily increasing proportion of CD4hi cases (CD4 count >500 cells/μl) and continuously rising rate of recent HIV-1 infections. In China, the overall improvement on immune status of HIV-1-infected persons can be partially explained by the scale-up of HIV testing. It was reported that between 2009 and 2018, the total person-times of HIV testing in China increased from 55.6 million to more than 240 million (Ding et al., 2019), which gives a chance to find more cases at the early stage of the disease. Moreover, young people seem to be more likely to know their HIV-1 status. A cross-sectional survey of HIV-1-infected MSM from seven cities showed that compared with old people, young Chinese MSM had a higher HIV-1 incidence and a higher prevalence of recent HIV-1 infection during 2012–2013 (Mao et al., 2018). Consistent with this finding, in the present study, we reported for first time that young MSM cases (< 30 years) in Heilongjiang Province exhibited better immune state and a higher recent infection rate than the old cases (≥ 30 years). The increase in number of young MSM cases was the positive factor and critical reason for the improved immune status and the elevated recent infection rate of MSM cases.

However, it is worth noting that recently infected HIV-1 cases are usually highly infectious because of their high viral loads, but they mainly remain undetected (Buskin et al., 2014). And, young MSM exhibited a higher prevalence of high-risk behaviors (recreational drug use, unprotected anal intercourse, and concurrent multiple sex partnerships) than old MSM (Mao et al., 2018). Therefore, recently infected young MSM have potential to speed up HIV-1 transmission and secondary infection within MSM population. We speculate that the rapid increase of young MSM cases with high CD4 counts will continue to be an important feature of HIV-1 epidemic in this northeast province of China in the following years.

One more evidence for the overall improvement of immune status of the HIV-1-infected persons was also observed in this study. As shown in Table 1, the proportion of “late diagnoses” (CD4 count <200 cells/μl at diagnosis) in newly diagnosed ART-naïve HIV-1 cases ranged from 20.7% to 23.1% during 2010–2016, much lower than the national level (35.5%–41.8%) in 2010–2014 (Jin et al., 2016), and the Heilongjiang provincial level in 1993–2012 (28.6%–38.4%) (Shao et al., 2014). This may be due to the high availability of HIV testing in China and people’s increasing willingness to test.

CRF01_AE genotype which accounts for only approximately 5% of global HIV-1 infections concentrates in Southeast Asia and China (Hemelaar et al., 2011, 2020). In 1990s, CRF01_AE virus was introduced into China and rapidly spread throughout the whole country. Since 2007, CRF01_AE became the most prevalent genotype in China except the northwest region (Feng et al., 2013; Li et al., 2017). Previous studies have reported that in Northeast China, more than half of HIV-1 infections were caused by CRF01_AE genotype (Li et al., 2016b), which is supported by our present findings. Here, we found that CRF01_AE was the main genotype in Heilongjiang in 2010, and continued to dominate HIV-1 genotype during 2010–2016. For HIV-1-infected MSM, CRF01_AE accounted for 64.3% (429/667) of newly diagnosed cases in Heilongjiang, slightly higher than the national level (57.36%) described in a meta-analytic integration of 66 molecular epidemiological studies conducted during 2008–2016 (Yin et al., 2019). That was why CRF01_AE-infected MSM mostly affected HIV-1 epidemic features among MSM cases.

In another study on HIV-1-infected MSM (aged 16–25 years) from 13 provinces in northwest, eastern, and southwest regions of China, the authors reported that the proportion of CRF01_AE infections decreased from 55.4% to 43.5% between 2009 and 2014 (Li et al., 2015b). In the present study, we added the data in Northeast China. Here the proportion of CRF01_AE infections among young MSM (aged <30 years) obviously increased from 2010–2011 (35.3%, 6/17) to 2012–2014 (66.7%, 42/63, *p* = 0.0191), and 2015–2016 (68.5%, 85/124, *p* = 0.0072). Based on our data, the young MSM infected with CRF01_AE virus increased disproportionately during 2010–2014 in Heilongjiang Province, Northeast China, and the proportion of this population remained stable thereafter. There was no sign that the proportion of this young MSM population would decline. In future, more concerns should be put on the measures of HIV-1 prevention and control targeting the young MSM with CRF01_AE infection.

There were limitations in this study. First, although we had made great efforts to collect samples from the clinical monitoring site, the biggest site in Heilongjiang Province, the sample size in early years (2010–2011) was still small (*n* = 140). Therefore, in some statistical analyses, differences among groups or subgroups may be underestimated. Even so, several important findings were yielded. We believe that using a larger sample size will further confirm our findings. Second, the data from cases that were diagnosed in very recent years lacked. In fact, we had collected the demographical and clinical information of HIV-1-infected persons who were diagnosed in 2017–2019 (*n* = 853). The median age of these cases was 33 years (IQR 27–45) and the proportion of young cases (aged <30 years) was 32.7% (279/853). This seemed that the younger age trend of local new HIV-1 infections was continuing. But because most of them lacked information on CD4 count, transmission route, and HIV-1 genotype, these cases were not included in this study. Third, HIV-1 genotyping based on partial *gag* and *env* gene regions might introduce some bias. Further analyses based on the near full-length genome of HIV-1 will be required in future. Despite these limitations, we still believe that our work could provide several clues for further research and HIV/AIDS control in Northeast China.

In summary, HIV-1 epidemic in Heilongjiang Province of China showed three new changes: the median age continued to decline, the cases with good immune status disproportionally expanded, and the recent infection rate steadily increased. MSM cases drove these changes. Rapid increase in the number of young MSM cases, mainly young MSM with HIV-1 CRF01_AE infection, was the most important contributor to these changes. Young MSM population has become a new hotspot and vulnerable group for HIV-1 transmission in this northeast province of China and development of intervention measures targeting this population is urgently needed.

## Data availability statement

The datasets presented in this study can be found in online repository. The name of the repository and accession numbers can be found in the article.

## Ethics statement

This study was approved by the Institutional Review Board of the Fourth Affiliated Hospital of Harbin Medical University ([2015]KT003). Written informed consent was obtained from each participant or participant’s legal guardian.

## Author contributions

F-XW and S-LL conceived the experiments. F-XW and Q-HL designed the experiments. X-HC collected the clinical samples. Q-HL, J-YW, S-YL, S-LZ, and W-BZ performed the experiments. J-YW, Y-QZ, E-LL, and Y-RW analyzed the data. Q-HL and J-YW wrote the original draft of the manuscript. F-XW and S-LL reviewed and edited the manuscript. All authors contributed to the article and approved the submitted version.

## Funding

This work was supported by the National Natural Science Foundations of China (grant numbers 81971915 and 81601755) and Natural Science Foundation of Heilongjiang Province (grant number C2017043). The funders had no role in the study design, data collection, analysis, decision to publish, or preparation of the manuscript.

## Conflict of interest

The authors declare that the research was conducted in the absence of any commercial or financial relationships that could be construed as a potential conflict of interest.

## Publisher’s note

All claims expressed in this article are solely those of the authors and do not necessarily represent those of their affiliated organizations, or those of the publisher, the editors and the reviewers. Any product that may be evaluated in this article, or claim that may be made by its manufacturer, is not guaranteed or endorsed by the publisher.

## Supplementary material

SUPPLEMENTARY FIGURE S1 Genotype identification of HIV-1 isolates from newly diagnosed participants. Ðhylogenetic trees were constructed with env (A), gag (B) sequences and corresponding subtype references, respectively, in Mega 6.06 software using the neighbor-joining method based on Kimura two-parameter model. Bootstrap analysis was conducted with 1000 replications. 07&08&C, virus that had a genotype of CRF07_BC, CRF08_BC or subtype C. The reference sequences on the clusters that contained newly identified sequences were marked as black dots, and the clusters without newly identified sequences were compressed and labeled by the subtype names. The branches colored in green in gag tree indicated sequences that contained an inter-subtype recombinant gag gene.
