# Supplementary figures and images for: Young MSM changed temporal HIV-1 epidemic pattern in Heilongjiang Province, China
Source: Front Microbiol. 2022 Nov 25;13:1028383. doi: 10.3389/fmicb.2022.1028383 (PMC9732660; doi:10.3389/fmicb.2022.1028383)

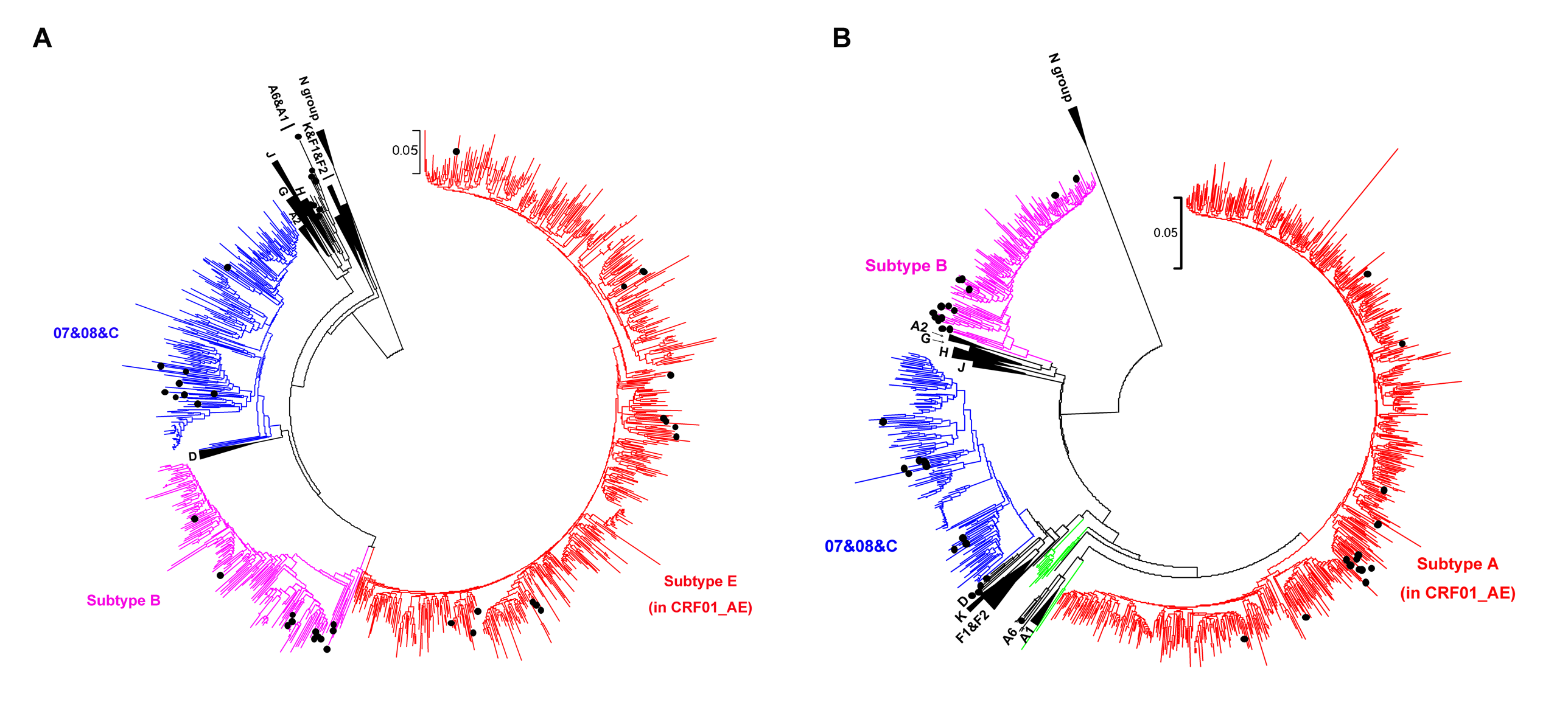

Supplement: SUPPLEMENTARY FIGURE S1 — Genotype identification of HIV-1 isolates from newly diagnosed participants. Ðhylogenetic trees were constructed with env (A), gag (B) sequences and corresponding subtype references, respectively, in Mega 6.06 software using the neighbor-joining method based on Kimura two-parameter model. Bootstrap analysis was conducted with 1000 replications. 07&08&C, virus that had a genotype of CRF07_BC, CRF08_BC or subtype C. The reference sequences on the clusters that contained newly identified sequences were marked as black dots, and the clusters without newly identified sequences were compressed and labeled by the subtype names. The branches colored in green in gag tree indicated sequences that contained an inter-subtype recombinant gag gene. [file Image_1.TIF]
