# Supplementary material for: Young MSM changed temporal HIV-1 epidemic pattern in Heilongjiang Province, China
Source: Front Microbiol. 2022 Nov 25;13:1028383. doi: 10.3389/fmicb.2022.1028383 (PMC9732660; doi:10.3389/fmicb.2022.1028383)
Supplement: Supplementary file 2 [file Table_1.docx]

**Table S1 Basic information of cases included and excluded in this study**

|  | Included cases  (n = 1006) | Excluded cases  (n = 177) | *P* value |
| --- | --- | --- | --- |
| Sex |  |  | 0.5882 |
| Male | 943 (93.7) | 164 (92.7) |  |
| Female | 63 (6.3) | 13 (7.3) |  |
| Age, median (IQR) | 35 (29-45) | 35 (28-45) | 0.6572 |
| Age groups |  |  | 0.4242 |
| <30 | 275 (27.3) | 57 (32.2) |  |
| 30-39 | 349 (34.7) | 52 (29.4) |  |
| 40-49 | 215 (21.4) | 36 (20.3) |  |
| >49 | 167 (16.6) | 32 (18.1) |  |
| CD4 count, median (IQR) | 384 (214-490) | 398 (231-489) | 0.7687 |
| CD4 count groups |  |  | 0.4976 |
| <200 | 227 (22.5) | 41 (23.2) |  |
| 200-350 | 194 (19.3) | 27 (15.2) |  |
| 351-500 | 345 (34.3) | 69 (39.0) |  |
| >5000 | 240 (23.9) | 40 (22.6) |  |
| Risk groups |  |  | 0.2841 |
| MSM | 667 (66.3) | 114 (64.4) |  |
| Heterosexual | 195 (19.4) | 44 (24.9) |  |
| Other groups | 12 (1.2) | 2 (1.1) |  |
| Unknown | 132 (13.1) | 17 (9.6) |  |

IQR, interquartile range; MSM, men who have sex with men; Other groups, risk groups including former plasma donors, injection drug users and mother-to-child infection. Data were shown as number (%) or median (IQR). The comparisons of the proportions were done by Chi-square test. The comparisons of age and CD4 count were done by nonparametric Mann-Whitney test.
