# Supplementary material for: Young MSM changed temporal HIV-1 epidemic pattern in Heilongjiang Province, China
Source: Front Microbiol. 2022 Nov 25;13:1028383. doi: 10.3389/fmicb.2022.1028383 (PMC9732660; doi:10.3389/fmicb.2022.1028383)
Supplement: Supplementary file 3 [file Table_2.docx]

**Supplementary Table 2 Basic information of heterosexually transmitted HIV-1 infections**

|  | Total  (n = 195) | Sampling year | | | *χ2* | *P* value |
| --- | --- | --- | --- | --- | --- | --- |
|  |  | 2010-2011  (n = 37) | 2012-2014  (n= 64) | 2015-2016  (n = 94) |  |  |
| Age (years old) |  |  |  |  | 5.70 | 0.4573 |
| <30 | 42 (21.5) | 5 (13.5) | 17 (26.6) | 20 (21.3) |  |  |
| 30-39 | 66 (33.8) | 14 (37.8) | 17 (26.6) | 35 (37.2 ) |  |  |
| 40-49 | 51 (26.2) | 13 (35.1) | 16 (25.0) | 22 (23.4) |  |  |
| >49 | 36 (18.5) | 5 (13.5) | 14 (21.9) | 17 (18.1) |  |  |
| CD4 count (cells/μl) |  |  |  |  | 7.56 | 0.2719 |
| <200 | 51 (26.2) | 10 (27.0) | 12 (18.8) | 29 (30.9) |  |  |
| 200-350 | 41 (21.0) | 12 (32.4) | 12 (18.8) | 17 (18.1) |  |  |
| 351-500 | 61 (31.3) | 10 (27.0) | 23 (35.9) | 28 (29.8) |  |  |
| >500 | 42 (21.5) | 5 (13.5) | 17 (26.6) | 20 (21.3) |  |  |
| HIV-1 Genotype (*gag/env*) |  |  |  |  | 4.95 | 0.5499 |
| CRF01_AE | 114 (58.5) | 22 (59.4) | 37 (57.8) | 55 (58.5) |  |  |
| Subtype B | 37 (19.0) | 9 (24.3) | 11 (17.2) | 17 (18.1) |  |  |
| 07&08&C | 33 (16.9) | 6 (16.2) | 13 (20.3) | 14 (14.9) |  |  |
| Subtype A & URF | 11 (5.6) | 0 (0.0) | 3 (4.7) | 8 (8.5) |  |  |
| HIV-1 infection status |  |  |  |  | 5.76 | 0.0561 |
| Recent infection | 62 (31.8) | 6 (16.2) | 25 (39.1) | 31 (33.0) | 5.76 | 0.0561 |
| Long-term infection | 133 (68.2) | 31 (83.8) | 39 (60.9) | 63 (67.0) | 5.76 | 0.0561 |

07&08&C, HIV-1 virus that had a genotype of CRF07_BC, CRF08_BC or C; URF, unique recombinant form. Both *gag* and *env* genes were used together to determine HIV-1 genotype of one sample. For the sample with only one gene available, HIV-1 genotype was determined by the known gene. Data were shown as number (%). The *P* values were calculated by chi-square test.
